# Detecting depression using an ensemble classifier based on Quality of Life scales

**DOI:** 10.1186/s40708-021-00125-5

**Published:** 2021-02-15

**Authors:** Xiaohui Tao, Oliver Chi, Patrick J. Delaney, Lin Li, Jiajin Huang

**Affiliations:** 1 School of Sciences, University of Southern Queensland, Toowoomba, Australia; 2 Advanced Analytics Institute, University of Technology, Sydney, Australia; 3 School of Computer Science and Technology, Wuhan University of Technology, Wuhan, 430070 China; 4 International WIC Institute, Beijing University of Technology, Beijing, 100124 China

**Keywords:** Major depressive disorder, Ensemble classification, Supervised machine learning

## Abstract

Major depressive disorder (MDD) is an issue that affects 350 million people worldwide. Traditional approaches have been to identify depressive symptoms in datasets, but recently, research is beginning to explore the association between psychosocial factors such as those on the quality of life scale and mental well-being, which will lead to earlier diagnosis and prediction of MDD. In this research, an ensemble binary classifier is proposed to analyse health survey data against ground truth from the SF-20 Quality of Life scales. The classifier aims to improve the performance of machine learning techniques on large datasets and identify depressed cases based on associations between items on the QoL scale and mental illness by increasing predictive performance. On the experimental evaluation on the National Health and Nutrition Examination Survey (NHANES), the classifier demonstrated an F1 score of 0.976 in the prediction, without any incorrectly identified depression instances. Only about 4% of instances had been mistakenly classified into depressed cases, with a significant accuracy of 95.4% comparing to the result from PHQ-9 mental screen inventory. The presented ensemble binary classifier performed comparably better than each baseline algorithm in all measures and all experiments. We trained the ensemble model on the processed NHANES dataset, tested and evaluated the results of its performance against mental screen inventory and discussed the comparable predictions. Finally, we provided future research directions.

## Introduction

Major depressive disorder (MDD) is one of the most prevalent healthcare issues worldwide [1]. According to the 2012 world health journal by the World Health Organization (WHO), more than 350 million people experience this illness, with many people having their quality of life impacted [2]. MDD has a range of different symptoms and at its most critical can result in suicidal thoughts, making it a global challenge for healthcare professionals for the past few decades. Depression can be successfully diagnosed by health experts when applying an operational diagnostic criteria of depression, such as a mental screening tool. However, an issue remains that a wide range of people with depressive symptoms do not seek clinic advice or professional care, resulting in healthcare professionals being unable to intervene in major cases of MDD [3].

In most situations, MDD is characterised by at least two weeks of developing low mood [4]. Therefore, early diagnosis of MDD is essential for early intervention [3]. A traditional approach to early diagnosis is the use of a self-assessment tool such as the Patient Health Questionnaire (PHQ-9), which is then followed up through a validation interview with a Mental Health Professional (MHP) [5]. The disadvantage of this process is that it may take time and incur costs, which is one of the barriers to patients with undiagnosed MDD seeking early assistance. Therefore, ways to automatically detect depressed cases in datasets may assist in early diagnosis of MDD and ensure depressed individuals engage with MHPs early.

Healthcare experts note that predicting depression can be based on lifestyle choices. The SF-20 Quality of Life Scale (QOLS) is an instrument used to measure association between chronic illness and lifestyle behaviours of people using survey data. These measures are based on the Mental Health Inventory (MHI-5), a short five-question instrument used to assess mental health [6]. Information from the MHI-5 can be checked against other contributing physiological and psychosocial factors to fully understand what contributes to low mood and mental disorders in an individual. To this end, QOLS contains conceptual categories that provide the basis for contributing factors, including material and physical well-being, relationships with other people, social, community and civic activities, personal development and fulfillment, and recreation [7]. While machine learning (ML) researchers have explored the link between these conceptual categories and other chronic diseases, ML-related research into the correlation between mental illness and other contributing factors is gaining attention. In a systematic review of ML techniques in mental health, Thieme et al. [8] found a growing application of multiple classification methods to structured datasets such as health questionnaires to detect depressive and mood symptoms using extracted features. The studies identified in their review apply techniques to data containing health history and previous behaviours, to more effectively distinguish between depressive and non-depressive persons. Mowery et al. [9] conducted a study into the association between depression and psychosocial stressors, which contained 12 categories ranging from demographic, socio-economic and environmental factors. These studies indicate that the need to further investigate the link between depressive symptoms and other factors that either cause or exacerbate them, which may provide a foundation for researchers and MHPs to increase their understanding of how we can predict MDD based on the physiological and psychosocial experiences of individuals.

Shatte et al. [10] identified two central themes in modern ML approaches, being the development of pre-screening diagnosis tools and the development of models to identify a person’s predisposition or risk for progressing to a severe mental health condition. Some of the most frequently reported challenges include robustly measuring and labelling mental health data as complex and dynamic due to noise or ambiguous data, generating low-dimensional features for reducing data into quantifiable categories for appropriate modelling and selection of models and training algorithms [8]. In studies involving ground truth especially, the challenge of low-dimensional labels can impact the accuracy of results. To this end, there is a need to improve the identification of appropriate features that can be used to help predict an individual’s disposition toward MDD [11]. One recommendation for improvement, being that future studies incorporate patient histories to improve the predictive capabilities of the diagnosis [10].

The National Health and Nutrition Examination Survey (NHANES) incorporates healthcare validation tools for measuring health status, such as the Patient Health Questionnaire (PHQ-9). The PHQ-9 tool is a 9-item screening instrument for measuring the severity of depressive symptoms from no depression to major depressive disorder. The PHQ-9 is the only integrated measurement for depression in the NHANES because it is simple, reliable and widely used in clinical evaluations [5]. Several studies have used NHANES data and the integrated PHQ-9 tool to explore correlations between depression and health issues, such as an investigation into the relationship between depression and low cholesterol [12], the association between MDD and obesity [13], the relationship between serum leptim and depressive episodes [14], and associations between blood folate concentrations in reproductive aged women and MDD [15]. To date, very few studies have used the NHANES to evaluate MDD against multiple psychosocial functionalities using ML techniques. Very little work has been done to incorporate psychological domain knowledge into the development of classifiers and no previous ensemble classifier approaches have used the NHANES dataset, with most ML techniques using it to explore physical disorders such as diabetes.

The objective of the present paper is to propose a suitable ML method to discriminate depression from collected health data for further interview diagnosis. The work done in this research builds an ensemble classifier using psychological domain knowledge from the SF-20. This ensemble classifier is applied to 98 features extracted from the NHANES healthcare dataset relating to mental health, which are used to explore associations between mental illness and items of health-related functionality on the QOLS: social, general, role, pain and physical functionalities.

The contributions of this research include:

- Development of an ensemble classifier using psychological domain knowledge from the PHQ-9 and SF-20.
- Improving predictive performance for MDD by incorporating 98 features extracted from the NHANES dataset.
- Further enhancing the utility of a ground truth technique similar to [5] to distinguish between depressive and non-depressive persons based on self-identification in health questionnaires.
- Using this ground truth technique with the classifier to demonstrate a higher accuracy of detecting depressed cases in a large healthcare datasets such as the NHANES.

After discussing the contributions of this work, the remainder of this paper will provide background to the study and introduce the new ensemble classifier. This paper will firstly discuss related work on ML techniques in diagnosing MDD and discuss the use of ensemble classifiers in this domain. It will then discuss the Research Objective, followed by the Approach. The Experiment is then detailed, followed by the Results and Discussion of the experiment. A conclusion will present suggested future work.

## Related work

### Machine learning techniques in MDD

Several previous studies have applied learning algorithms to detect MDD symptoms on datasets containing patterns of behaviour among individuals, with the most common being [1]: Support Vector Machine algorithm [16–18], Naive Bayes method [19] and Random Forest technique [20]. In their systematic review of ML techniques in the mental health domain, Shatte et al. [10] identified regression and Decision Trees (DT) as other common approaches used. Along with KNN, these methods comprise typical representative techniques in machine learning experiments.

Predictive modelling for MDD symptoms on large datasets is a relatively new approach. Choudhury et al. [16] developed a probabilistic model to train crowdsourcing Twitter posts and develop a social media depression index to characterise the levels of depression in a sample population. The study used a SVM classifier with an RBF kernel for identifying depressive instances. Fivefold cross-validation was used to validate the performance of the classifier, with results yielding an average accuracy of 73% and high precision of 82% [16]. The depression index had a strong correlation with national depression statistics [16]. Importantly, this approach established the need to add social environment and external factors to MDD assessment.

Similarly, Tsugawa et al. [17] built a SVM supervised learning model to use features from online tweet activities to predict users’ current depression status. Features used for predicting depression were extracted from the activity history of users. In this approach, an accuracy of 69% can be reached through the prediction of depressive users by the proposed classifier [17]. The trusted status (critical standard) of users were generated by CES-D and BDI screening scales of all participants. The limitation of this study found that long observation periods for collecting data may decrease accuracy [17, 21].

In combining a Random Forest (RF) algorithm and SVM technique, Fatima et al. [20] were able to discriminate depressive posts and communities from non-depressive posts and communities in the online social network Livejournal. LiveJournal enables users to provide pre-defined “mood tags” on user posts, which were extracted features to measure depression levels among users who created posts and participated in communities. The study implemented Random Forest algorithm with SVM classifier for text classification to find the maximum margin between severe depressed, moderate depressive and non-depressed classes. In the experiment, RF performed better in comparison with a standard SVM method, as the proposed model achieved about 90% and 95% accuracy in classifying the depressive posts and depressed communities, respectively [20].

What many of these previous studies have in common is that they use single ML techniques and often use text-based data, such as datasets from social media, to predict MDD. Applying multiple techniques might improve the precision of detecting depressive symptoms in a wider range of data. The prior studies reviewed in this section adopt mainstream techniques such as DT, ANN, KNN and SVM as methods for machine learning experiments. As such, we can use these as typical representative techniques for baseline solutions as part of the comparative analysis against our ensemble classifier.

### Ensemble classifiers

Instances of ensemble classifiers have proved promising in accurate diagnosis, particularly when using multiple features. Hassan et al. [19] used majority vote for classification and regression on top of predictions from three single classifiers: SVM classifier, Naive Bayes (NB) classifier, and Maximum Entropy (ME) classifier. The study illustrated how to find individual depression scale by observing and extracting emotions as features from text on different social media platforms [19]. The performance accuracy of SVM is 91%, 83% and 80%, respectively, for NB and ME classifiers. In another study exploring data from Chinese social media network Weibo, Peng, Hu and Dang [18] used a multi-kernel SVM-based model on three categories of features, user microblog text, user profile and user behaviours [18]. The multi-kernel SVM method had a lowest error rate 16.5% for identifying depressed people [18]. This study demonstrated that an ensemble method can obtain better predictive performance using multiple learning algorithms than single traditional learning algorithms alone [18].

Such studies apply ensemble methods to rich textual data from social media, which is one of the most common applications of these approaches [8]. The use of survey data such as health questionnaires is gaining precedence in studies, as the process of feature extraction can be useful in correlating external factors with possible MDD outcomes. Yang and Bath [22] built an ensemble classifier for predicting depression in the elderly, with different models (GBM, KNN, RGF and LR) used to explore different factors such as demographic, social engagement, physical health and disability, psychological and mental health, lifestyle and cognition factors. This improved predictive performance and provided greater insight into risk factors that could lead to severe depression in older adulthood, assisting early intervention. A study by Srividya et al. [23] developed an ensemble classifier to identify mentally distressed individuals in a target population of high school students and working individuals, with an accuracy score of 90%. Their study explored the association mental distress against education, socio-economic, life satisfaction and relationship quality factors, with the researchers indicating that the incorporation of additional parameters (e.g. physiological) could be added to extend predictive models to a range of other mental illnesses such as MDD. To date, studies have applied ensemble classifiers to mental illnesses such as stress [11] and internet addiction [24], with only a handful having applied it to depression [22].

For better predictive modelling, researchers need to incorporate additional parameters into an ML classifier. This leads to the challenge of both reducing the dimensionality of features and the selection of appropriate analytics techniques, which can each be affected by the quality of data collection [1]. These prior studies have employed multiple classification techniques, but there is still a need to test additional techniques for detecting depression, particularly in using large and varied samples. However, there is a lack of research into an efficient machine learning classifier for detecting depression in large data. The study by Hsieh et al. [24] utilised an ensemble classifier on internet addiction, but applications of this method to depression are in their infancy. These prior studies have incorporated parameters such as physiological, but there has been a small amount of work that uses psychological domain knowledge to build ensemble classifiers. Furthermore, few studies have used ground truth in combination with ensemble classification in this area. One study developed a ground truth dataset to test an ensemble classifier on mental health instances, but again, this study used social network data [25]. In [26], they used an ensemble classifier with ground truth to predict happiness based on a range of different data including physiological and behavioural, demonstrating the feasibility of the approach suggested in this research.

This work attempts to use psychological domain knowledge such as that of the SF-20 QOLS [5]. Only a handful of studies have used ensemble classifiers on survey questionnaires, as indicated in a recent systematic review [8], with none using the NHANES data. This dataset is mostly used for physiological studies, however, the survey contains notable health-related variables that can influence mental health. To the best knowledge of the authors, this is the first study to attempt to use the NHANES data in such a work.

## Research definitions

This research aims to design an effective ensemble classifier method for automatically detecting depressed cases in healthcare datasets. The objective is to develop the classifier based on psychological domain knowledge and use a process of ground truth to measure features in the NHANES survey data that are related to the functioning categories in the SF-20 QOLS.

To outline the objectives of the research, the first definition is:

### Definition 1

Let $\mathbb {S}$ be a set of user properties to present an effective user profile for depression, a user property s $\in$ $\mathbb {S}$ is a tuple $s := \langle p_{1}, p_{2}, p_{3}$, $\cdots p_{n} \rangle$, where

- *p* is a visualisation or instance of an user property;
- *p* is not a mental or depression closly related symptom;
- *n* could be an infinite integer so the number of *p* elements could be unlimited;
- All *p* elements in the same user profile are generally independent.$\square$

With clear definition of research objective, the research target is defined as:

### Definition 2

Let $\mathbb {V}$ be a set of labeled user depression, a label of user depression $v\in$ $\mathbb {V}$ is a screening result of personal depression, where

- When *v* is binary, it presents depression (1) or healthy (0);
- When *v* is scale, it presents the severity of depression from healthy (0) to most severe (1).$\square$

From Definition 1, any given user property $s \in \mathbb {S}$ is possibly overlapped with other user properties. While learning from related psychological researches, a set of user personal functionalities can present a reflection of a user’s mental profile. This method can potentially detect depression by analysis of a set of user functionalities. Therefore, given the definition below:

### Definition 3

Let $\mathbb {U} = \langle u_{1}, u_{2}, u_{3}$, $\cdots u_{k} \rangle$ be a sub-set of $\mathbb {S}$, any element u $\in \mathbb {U}$ is a tuple $u := \langle p'_{1}, p'_{2}, p'_{3}$, $\cdots p'_{n'} \rangle$, where

- $\mathbb {U}$ is a machine learning descriptive sub-set transferred from $\mathbb {S}$ in psychological domain descriptive;
- $\forall p'\in$ u is assigned from an instance $p\in a$ in Definition 1;
- $|\mathbb {D}^s|$ is limited due to the small functionalities defined in psychological domain.$\square$

The research problem can be defined as an effective classification model $\mathbb {M}$ that provides a reliable mapping function for a well-defined $\mathbb {U}$ to map into $\mathbb {V}$:

$$\begin{aligned} \mathbb {U} \,{\mathop {\Rightarrow }\limits ^{{\mathbb {M}}}}\, \mathbb {V}\hbox { or }\mathbb {M}(\mathbb {U}) = \mathbb {V}. \end{aligned}$$

Generally, we can label the cases waiting for detection into two classes: depression instances and non-depressed instances. The binary classification is seen as supervised learning because the objective is to use machine learning to automatically classify participants into two labelled categories of depression and non-depression.

## Approach

### NHANES survey data

In this study, we use the dataset from the National Health and Nutrition Examination Survey (NHANES). NHANES is designed to collect health-related information about the U.S. household population. It is a rich data source for health professionals and researchers for various modern health problems. It is conducted by the National Center for Health Statistics (NCHS), which is part of the Centers for Disease Control and Prevention (CDC). All information in NHANES is gathered and protected with the requirement of Federal Law of U.S. and for health research purposes only. Collections of NHANES in the last decade are free for researchers and published on the website of the NCHS.

We employ the questionnaire data in NHANES 2013–2014 collection as input data $\mathbb {H}$ for the experiment. The age of participants is set to 18+, because data for teenagers and children are only partially published. As our objective is to classify general individuals into non-depressive and depressive groups, the features only involved with a single gender are excluded.

### Build ground truth

Using the integrated PHQ-9 screen measurement, we can establish ground truth label information on whether or not a participant has depression) for the whole dataset. The PHQ-9 measurement scales contain five levels of depression severity, from minimal-level to severe-level. In the research of Kroenke et al. [5], patients who were identified at the moderate-level (score $\ge$ 10) of depression in the PHQ-9 measurement had a sensitivity of 88% and a specificity of 88% for MDD. We therefore choose the separation at PHQ-9 score 10. Participants with a PHQ-9 score less than 10 are considered non-depressed and vice versa. We label these non-depressed people as the logical truth or “1”; conversely, those depressive people are labeled as the logical false or “0”.

We rationalise our classification of study respondents into depressive and non-depressive groups because our ground truth is based on their self-reporting in the PHQ-9 component of the NHANES dataset. Given that our profile of study respondents is based only on their self-identification in the survey and not on clinical diagnosis of these individuals by medical professionals, we cannot separate them into different levels of depression (i.e. severe to minimal). Without the clinical diagnosis and their medical reports, we are dependent on their self-reporting of their state of depression using the tool to be reliable, hence we use the standard of the two-classification approach employed by [5]. Additionally, to predict mental status, we do not include their feelings and expressions in the features.

### Conceptual framework

The conceptual framework is the theoretical structure encompassing all level models and classification methods. In this study, the framework consists of three layers:

1. Psychological domain knowledge transfer;
2. Data processing;
3. Classification Modelling.

**Fig. 1:**
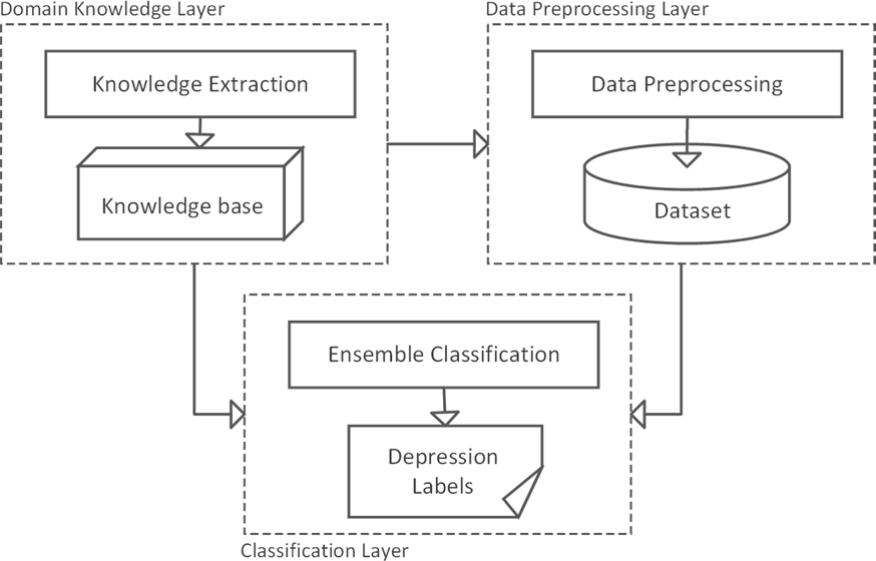
Conceptual framework

The conceptual design of the framework is illustrated in Fig. 1.

The framework is implemented in the research experiment. The psychological knowledge layer learns how to group health informatics in the psychological domain. It directs the actions for the data processing layer to transform the dataset. It also assists designing the ensemble classification technique in the classification modelling layer. The data processing layer contains all proceedings of data preprocessing, feature extraction and establishment of the dataset. The layer converts the health statistics dataset into several normalised datasets ready for classification. The classification layer implements the classification of dataset. It builds an effective ensemble classifier and performs the comparative prediction of depressive risk for participants.

#### Psychological knowledge

This layer is informed by the PHQ-9 instrument and the SF-20 QOLS. The PHQ-9 measures the level of depression severity, while the SF-20 contains questions around health-related functionalities related to quality of life. Kroenke et al. [5] discovered a strong association between increasing depression severity screen scores on the PHQ-9 and worsening functionality on all 6 categories of the SF-20 Quality of Life scale. The 6 categories are mental, social, role, pain, physical and general functions. Five items are derived from health diagnostic criteria in the Mental Health Inventory (MHI-5) and the sixth classifies mental disorder symptoms as mental category.

**Fig. 2:**
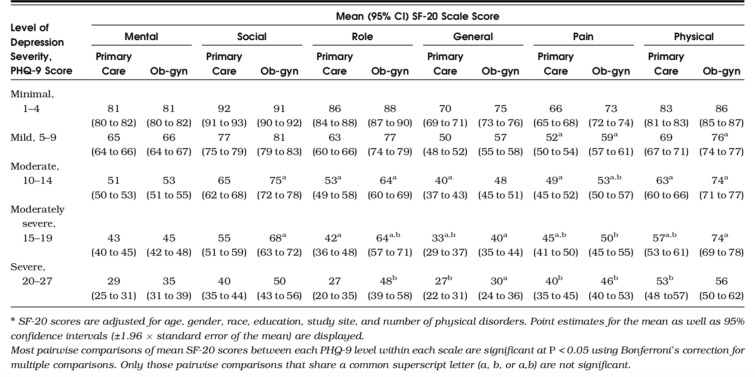
The relationship between depression severity and personal health-related functionalities [5]

Associations of health functionalities with MDD have been observed in many previous studies. In research by Clark et al. [27], they examined the opposite association of depression and psychosocial functionalities. Using 5 domains of physical, social, emotional, cognitive, and spiritual functioning. It found that depression is associated with poor health status and negative health behaviours. This layer intends to implement a similar approach with the health-related functionalities of the SF-20. Figure 2 from [5] illustrates the relationship between increasing PHQ-9 scores of depression and worsening functional categories (see Fig. 2).

The relationship between MDD and the scales of health-related functionalities have the similar trend as the severity of depression in statistics. Previous studies in related-work focused on detecting depressive symptoms and depression-related contents. Likewise, the relationship used in the implementation here presents a new potential method of predicting users’ depression by sampling various diagnostic criteria of functionality. The combination of the classification technique and the binary ground truth technique could potentially enhance the strength of new predictive approaches. This new method has the following advantages compared to previous techniques:

- There are more features available for classification due to enlarged inputs in various functional areas;
- It is easier to acquire functional data than sensitive data of depressive symptoms, especially on social networks;
- It is easier to cover sufficient specificities of one functional status than to cover all available types of depressive symptoms;
- Using six functional status groups rather than only one collection of depressive symptoms will produce more accurate and comparable classification.

Therefore, we can apply psychological domain knowledge to the information domain. $\mathbb {D}^s$ can be leveraged and divide into 6 sub-datasets. The dataset of user mental profile needs to be redefined:

##### Definition 4

Let new redesigned $\mathbb {U}=\langle u_{m}, u_{s}, u_{r}, u_{pa}$, $u_{ph}, u_{g} \rangle$, in which every $u\in \mathbb {U}$ is an independent function of a user where

- $u_{m}$ is an individual mental disorder symptom;
- $u_{s}$ is a diagnostic criteria in the social activities;
- $u_{r}$ is a diagnostic criteria in the role functionality;
- $u_{pa}$ is a diagnostic criteria in the pain domain;
- $u_{ph}$ is a diagnostic criteria in the physical category;
- $u_{g}$ is a diagnostic criteria in the general actions.

$$\square$$

#### Data processing

The survey questions in the NHANES dataset are spread across columns and participants are placed into rows, separated into different tables of health domains. Since the tables are not organised in the same format and structure, preprocessing is required for classification in the experiment. We will only use the survey questions component, which is one third of the dataset.

**Fig. 3:**
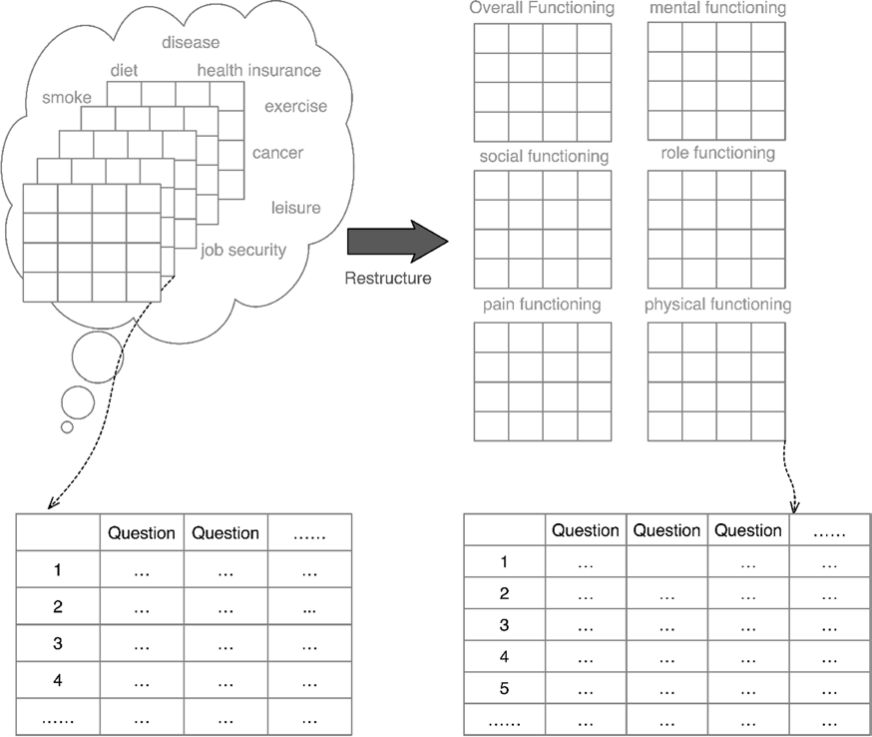
Data restructure based on psychological knowledge

Data cleaning and transformation was used to eliminate redundancies and ensure the data is computer-readable. The data types are justified to make the dataset compatible and comparative. Normalisation was also necessary to uniform the scale condition in various questions. Whilst data preprocessing is implemented, the psychological domain knowledge in functional diagnostic criteria is applied in the reconstruction of data structure. According to Definition 4, we can lower the dimension of data set by reducing the number of tables. All tables need to be reconstructed into only six tables referred by six categories of depression diagnostic criteria in functionality (see Fig. 3). They may involve different number of questions but they all have the same participants. Furthermore, those six tables can be rejoined into one table due to same row index. By instant consideration of those tables, each table forms a new dataset where participants are cases and questions are features. We can therefore define the new datasets after data preprocessing as below:

##### Definition 5

Let new overall dataset of *m* cases and *n* features $\mathbb {D}_{o}=\displaystyle \big \{ (x_{1}, x_{2}, ... , x_{n}, y), x_{i} \in R^{m}, y \in {\{0, 1\}}^{m} \big \}$, and sub-datasets of different six functional categories $\mathbb {D}_{m}$, $\mathbb {D}_{s}$, $\mathbb {D}_{r}$, $\mathbb {D}_{pa}$, $\mathbb {D}_{ph}$ and $\mathbb {D}_{g}$, where

- $$|\mathbb {D}_{o}|=|\mathbb {D}_{m}|=|\mathbb {D}_{s}|$$ = $$|\mathbb {D}_{r}|$$ = $$|\mathbb {D}_{pa}|$$ = $$|\mathbb {D}_{ph}|$$ = $$|\mathbb {D}_{g}|$$ = *m*;
- The space of features in $\mathbb {D}_{m}$, $\mathbb {D}_{s}$, $\mathbb {D}_{r}$, $\mathbb {D}_{pa}$, $\mathbb {D}_{ph}$, $\mathbb {D}_{g}$ = the space of features in $\mathbb {D}_{o}=n$.

$$\square$$

#### Classification modelling

We use an ensemble classification approach to build the model. It implements the independent ensemble methodology which applies several classification techniques in parallel. The model implements Support Vector Machine (SVM) technique, Artificial Neutral Network(ANN) algorithm, K-Nearest Neighbour (KNN) method and Decision Tree (DT) method. Each composite classifier among them is trained on the same portion of the training set in one run.

The performance of the approach is evaluated by a k-fold cross validation-algorithm. By amalgamating all outputs of composite classifiers into a single prediction, we generate the ensemble classifier. This ensemble classification approach collects various outputs of multiple independent classifiers and combines them to improve the predictive performance.

In general, an ensemble method provides higher accuracy and better predictive performance than a single algorithm [28]. Several advantages in performance include [29]:

(i) *Overfitting avoidance* by averaging different hypothesis to reduce the risk of choosing an incorrect hypothesis.
(ii) *Computational advantage* in decreasing the risk of obtaining a local minimum by combining several learning ensemble methods.
(iii) *Strong representation* in achieving a better fit to the data space by combining different models and extending the search space.

Moreover, ensemble methods are considered the potential solution for several machine learning challenges like class imbalance, concept drift and curse of dimensionality [29]. The ensemble method also imitates human nature by seeking various solutions before making a final decision. The ensemble method for this experiment is considered an optimised technology comparing to other baseline models in the classification of our preprocessed data.

### Ensemble model

The integration of four methods is expected to optimise predictive performance. As each independent sub-model is trained, the ensemble classifier covers more target concepts. To combine all baseline classifiers outputs, our modelling procedure adopts the weighting ensemble method. Weighting ensemble method is very generic when all base classifiers have uniform comparable outputs. The weight of each classifier can be set proportional to its accuracy performance on a validation set [28]:

1 $$\begin{aligned} w_{i} = \frac{1 - E_{i} }{\sum _{k = 1}^{n} (1 - E_{k}) }, \end{aligned}$$

where $E_{i}$ is a normalisation factor based on the predictive performance of classifier *i* in the validation set.

Because the ensemble classifier combines the weighted outputs of all base classifiers, we can define the ensemble classifier as below:

#### Definition 6

Let the ensemble model

2 $$\begin{aligned} \mathbb {M}_{e} = \sum _{k = 1}^{n} w_{i} M_{i}, \end{aligned}$$

where

- $M_{i}$ presents a single base model;
- $w_{i}$ presents the weighting metric of predictive performance at specific base model $M_{i}$;
- *k* is the order of base models;
- *n* is the total number of base models, and in our case $n = 4$;
- *i* is the order number of specific base model.$\square$

The principle of this ensemble approach is to build estimators independently and then find the average of their predictions. The combined estimator is usually better than any single base estimator because instances variance is moderated.

### Adapted classification methods

Our ensemble classification method involves several baseline supervised classification models, consistent with predictive data mining. We selected supervised learning algorithms with diverse advantages. Each classification method has a diverse computing algorithm, with the goal being to build a concise model to achieve the best possible prediction accuracy. The supervised machine learning techniques used include [30]:

- Logic-based algorithm: the algorithms use logic or rules to make a decision of selecting proper features during the learning. DT method adopts this algorithm.
- Perceptron-based techniques: the algorithms are based on the notion of perceptron to construct pattern-like layers of neutrons to learn different paths in the classification. Neutral network is its well-known representer.
- Statistical learning algorithms: the algorithm uses statistical approaches to provide a probability that an instance belongs in each class. Under this category of classification algorithms are Naive Bayesian network and k-Nearest Neighbour technique.
- Support vector machines: SVM uses a hyperplane to separate two data classes and the margin created by the separating hyperplane indicates the success of the classification [30].

### Baseline models

The choice of a suitable algorithm depends on the type of problem and the given data, and the accuracy can be improved by using two or more algorithms together [31]. In Section 2.1, a number of prior studies were discussed who implemented several typical representative ML techniques to analyse data. The techniques are taken as mainstream standards with the studies cited being contemporary and exploring similar work to what is demonstrated here. Hence, the ML techniques identified in Section 2.1 are the baseline models for our experiment. We thus propose one method of each type to present sufficient algorithms in the limited number of sub-models, selecting four techniques as our baseline models: Decision Tree method (DT), Artificial Neutral Network technique (ANN), k-Nearest Neighbour (KNN) method and Support Vector Machine (SVM) algorithm.

Given a well-preprocessed dataset of m examples and n features $\mathbb {D}$ = $\displaystyle \big \{ (x_{1}, x_{2}, ... , x_{n}, y ), x_{i} \in R^{m}, y \in {\{0, 1\}}^{m} \big \}$, we can generate a suitable ensemble model $\mathbb {M}_{e}$ to present a mapping of $\big \{ x_{1}, x_{2}, ... , x_{n} \big \}$ to $\big \{ y \big \}$ by applying *h* various types of baseline model $M_{i}$: 
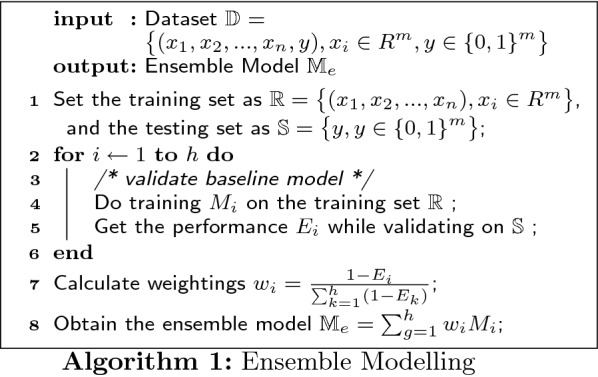


## Experiment

We employ an ensemble supervised learning experiment to classify depressive users in the health survey dataset $\mathbb {H}$. Using psychological knowledge, we reduce the dimension of the dataset by splitting it into sub-sets. This will benefit the processing of classification, while comparing the performance of the overall dataset and sub-sets for support of further solution on the real condition with less features.

### Data preprocessing

The NHANES dataset contained errors and missing values. Not all questions were completed by participants. Further to this, the questionnaire involves “Refuse” and “Don’t Know” options for nearly every question due to considerations of personal privacy and the right not to disclose. Data preprocessing involved filling, correcting and normalising these inputs to prepare it for the experiment. In order to uniform all actions taken in data cleaning, we design a couple of presumption and principles to manage the proceeding:

- We assume that missing inputs belong to the persons who have on depressive risk;
- The choice of “Refuse” option or “Don’t Know” option is presumed normal which can be corrected by the statistical mean of inputs;
- All inputs of survey questions should be converted into binary, range and numbers due to the design of answer options;
- The final value of each input should be normalised and have a limited byte size.

After preprocessing, an overall dataset was produced with a total of 5398 participants. Among them, 516 ( 9.56% ) are depressive persons and 4882 ( 90.44% ) are non-depressive. The features of the dataset are variables representing a major question in the NHANES survey. After rejecting several irrelevant major questions, a total of 98 features were produced. Among them, inputs in 49 features are binary, 36 features consists of range data and the remaining 13 features are float numbers. Grouping 98 features into separate functionalities by Definition. 4, we generated six sub-datasets (see Table 1).

**Table 1:** Features and sub-datasets

Dataset	$\mathbb {D}_{o}$	$\mathbb {D}_{ph}$	$\mathbb {D}_{r}$	$\mathbb {D}_{s}$	$\mathbb {D}_{m}$	$\mathbb {D}_{pa}$	$\mathbb {D}_{g}$
Features	98	7	9	6	4	2	70

### Experiment design

**Fig. 4:**
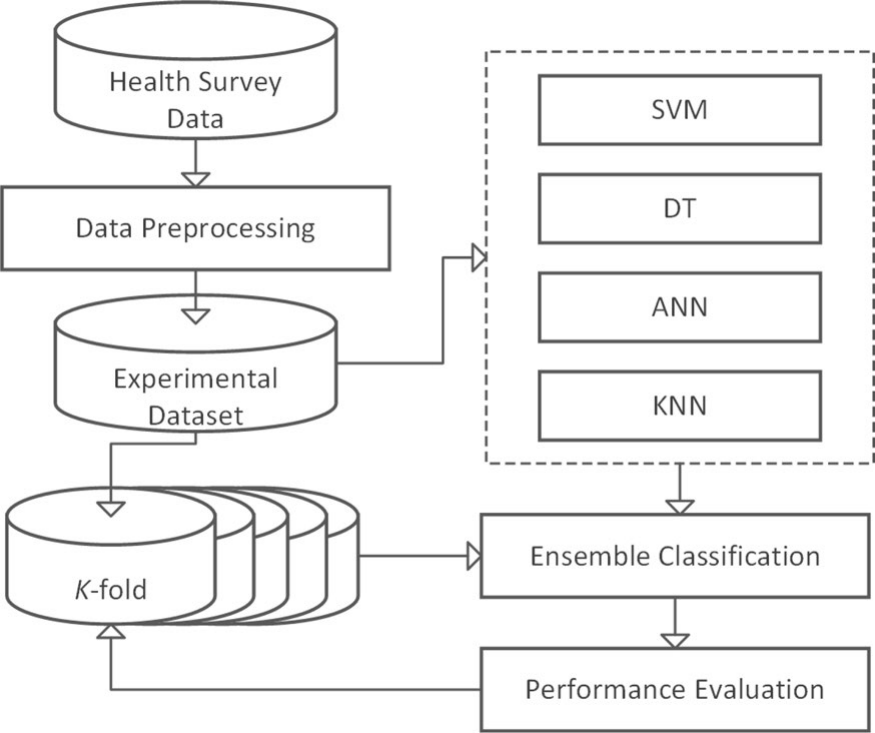
The experiment procedure

In the experiment, we first obtain dataset $\mathbb {D}_{o}$ by data preprocessing on survey data $\mathbb {H}$; next, we aggregate all features of $\mathbb {D}_{o}$ into 6 health-related functional classes and follow the same procedure to divide $\mathbb {D}_{o}$ into 6 sub-sets $\mathbb {D}_{ph}$, $\mathbb {D}_{r}$, $\mathbb {D}_{m}$, $\mathbb {D}_{s}$, $\mathbb {D}_{pa}$ and $\mathbb {D}_{g}$; and we train dataset $\mathbb {D}_{o}$ by four baseline models (DT, ANN, KNN, SVM) to obtain the relevant performances; then we build the ensemble model $\mathbb {M}_{e}$ by calculating the performance weight $w_{i}$ of each baseline model $M_{i}$; furthermore, we train all 6 sub-datasets by the ensemble classifier $\mathbb {M}_{e}$; and the final step is to use a k-fold cross-validation algorithm to determine the value of the complete predictive performance. The experimental procedure is depicted in Fig. 4.

From the proceeding details of classification, we can define the algorithm of whole experiment as below (see Algorithm 2):

The ensemble classification can be expressed in algorithm as well: Given a well-preprocessed dataset of m examples and n features $\mathbb {D}$ = $\displaystyle \big \{ (x_{1}, x_{2}, ... , x_{n}, y ), x_{i} \in R^{m}, y \in {\{0, 1\}}^{m} \big \}$, we can obtain the ensemble classifier $\mathbb {F}_{e}$ = $w_{svm} \cdot f_{svm}$ + $w_{nb} \cdot f_{nb}$ + $w_{knn} \cdot f_{knn}$ + $w_{dt} \cdot f_{dt}$ by applying supervised learning on dataset $\mathbb {D}$:

Many machine learning packages and tools are accessible to implement common classification algorithms. The scikit-learn library from Python provides simple and efficient tools for data mining and data analysis. And it nearly contains all supervised learning methods for both binary and multi-class classification. We thereby choose Scikit-learn Python package to implement four baseline models. 
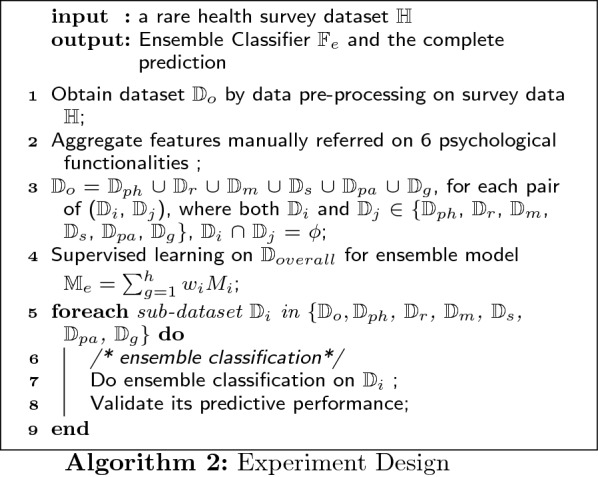


#### Kernel and parameters

Selecting suitable kernel and parameters is a common task for classification but it is also complex for specific examples. We only balance the settings of baseline models instead searching a perfect for the parameter because it is uncertain if the settings could maximum the performance in utter instances. And the predictive performance is expected to be improved by ensemble classification. We thereby employ common values for kernel and parameters. All four sub-models are configured for binary classification and their predictive performances are weighted in both labelled classes.

### Performance measure

The predictive performance of each base classifier in our model is evaluated by F1 score, which is generated on a confusion matrix of validation. In the confusion matrix, we simply set the number of real non-depressive cases in the training set as *condition positive (P)* and let the number of real depressive cases in the training set as *condition negative (N)*. F1 score is a balanced measure of both the precision (PPV) and the recall (TPR) of the validation:

3 $$\begin{aligned} F1 = \frac{2 }{\frac{1}{TPR} + \frac{1}{PPV}} = \frac{2TP}{2TP + FP + FN}. \end{aligned}$$

## Results and discussion

### Experimental results

F1 score is a weighted harmonic mean of precision and recall, such that the best score is 1.0 and the worst is 0.0. F1 measure equally considers both precision and recall in the performance measurement. We use F1 measure for the main indicator of model’s performance. According to Eqs. (1) and (2), we can calculate the weight for each base model (see Table 2) and further generate the complete form of ensemble classifier:

4 $$\begin{aligned} \mathbb {F}_{e} = 0.228 \cdot f_{svm} + 0.283 \cdot f_{nb} + 0.266 \cdot f_{knn} + 0.223 \cdot f_{dt}. \end{aligned}$$

**Table 2:** Performance and weights for sub-models

Models	Accuracy	F1 score	1 - F1	Weight
SVM	0.921	*0.958*	0.042	0.228
ANN	0.905	0.948	0.052	*0.283*
KNN	0.908	0.951	0.049	0.266
DT	*0.925*	*0.959*	0.041	0.223


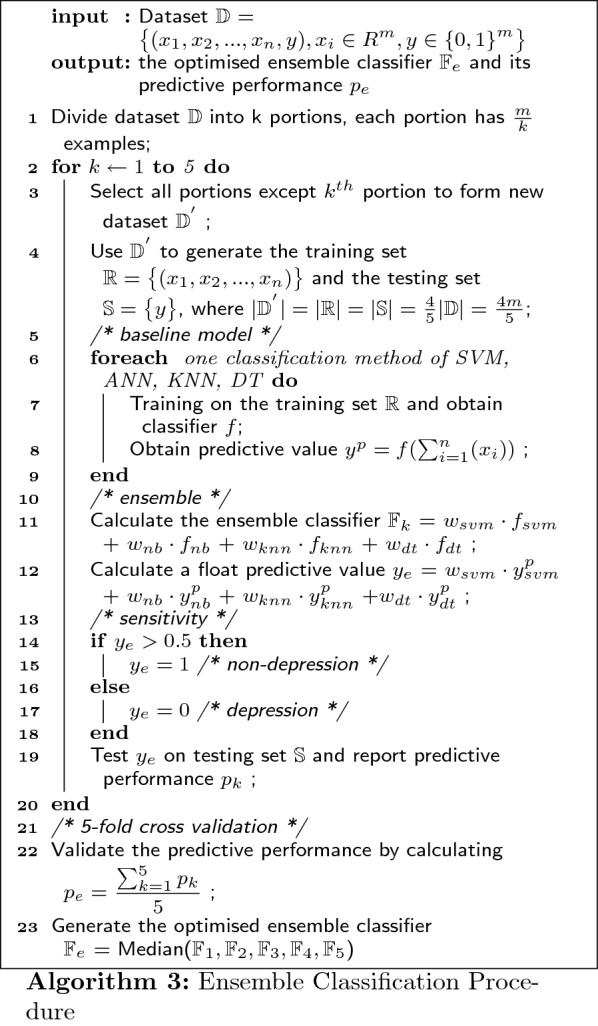


Accuracy indicates the number of correct predictions made in all occurrences of both labels. It presents all corrected predictions based on the results of the PHQ-9 mental screen inventory. Precision is the ability of a classifier not to label an instance positive that is actually negative. Here, we measure how effectively the model can diagnose a person’s psychological health. Recall is the ability of a classifier to find all positive instances. It measures how many non-depressed people are correctly identified. As the features and specificity of the overall dataset and each sub-datasets varied, the divided performances are expected (see Tables 3, 4 and 5).

**Table 3:** Features and performances of the ensemble classifier

Dataset	F1 score	Accuracy	Precision	Recall
$\mathbb {D}_{o}$	*0.976*	*0.954*	*0.956*	*1.000*
$\mathbb {D}_{ph}$	0.964	0.931	0.934	1.000
$\mathbb {D}_{r}$	0.963	0.929	0.929	1.000
$\mathbb {D}_{s}$	0.964	0.931	0.931	1.000
$\mathbb {D}_{m}$	0.975	0.953	0.960	0.999
$\mathbb {D}_{pa}$	0.961	0.925	0.925	1.000
$\mathbb {D}_{g}$	0.964	0.931	0.930	1.000

**Table 4:** Performances in F1 score

Models	$\mathbb {D}_{o}$	$\mathbb {D}_{ph}$	$\mathbb {D}_{r}$	$\mathbb {D}_{s}$	$\mathbb {D}_{m}$	$\mathbb {D}_{pa}$	$\mathbb {D}_{g}$
SVM	0.958	0.950	0.950	0.950	0.957	0.950	0.951
ANN	0.948	0.944	0.935	0.942	0.961	0.950	0.930
KNN	0.951	0.947	0.945	0.944	0.958	0.938	0.949
DT	0.959	0.950	0.949	0.950	0.960	0.950	0.950
Ensemble	*0.976*	0.964	0.963	0.964	0.975	0.961	0.964

**Table 5:** Performances in accuracy

Models	$\mathbb {D}_{o}$	$\mathbb {D}_{ph}$	$\mathbb {D}_{r}$	$\mathbb {D}_{s}$	$\mathbb {D}_{m}$	$\mathbb {D}_{pa}$	$\mathbb {D}_{g}$
SVM	0.921	0.904	0.904	0.904	0.919	0.904	0.907
ANN	0.905	0.895	0.879	0.892	0.928	0.904	0.873
KNN	0.908	0.900	0.896	0.895	0.923	0.886	0.904
DT	0.924	0.905	0.904	0.905	0.926	0.904	0.906
Ensemble	*0.954*	0.931	0.929	0.931	0.953	0.925	0.931

The ensemble classifier performed better compared to the baseline models, including an F1 score of 0.976 vs 0.959 achieved by DT, and an accuracy of 0.954 vs 0.924, which was the again achieved by DT. Performances in functionality sub-sets is compromised in this experiment, but is still comparable to other machine learning methodologies [18–21].

The results in Table 3 indicate that for the mental functionalities sub-set, the F1 score (0.975) and accuracy (0.953) is closest to the scores for the overall dataset. The accuracy and F1 scores in physical, social and role functionalities in isolation are below the performance of the overall dataset. This indicates that mental functionalities are most relevant to the classifier. $\mathbb {D}_{m}$ had 4 features, which suggests that these are the most relevant features to the psychological knowledge used to underpin the method. This is consistent with domain knowledge.

The prediction performance in the mental functionality sub-set shown in Table 5 is close to the whole dataset even though it has less features involved. This may indicate that features for mental functionality are more depression-related than features in other categories, because non-criteria items in the depression scale decreased in specificity of performance [4].

**Table 6:** Performances in recall

Models	$\mathbb {D}_{o}$	$\mathbb {D}_{ph}$	$\mathbb {D}_{r}$	$\mathbb {D}_{s}$	$\mathbb {D}_{m}$	$\mathbb {D}_{pa}$	$\mathbb {D}_{g}$
SVM	0.990	1.000	1.000	1.000	0.996	1.000	0.999
ANN	0.955	0.970	0.961	0.969	0.985	1.000	0.937
KNN	0.993	0.981	0.986	0.977	0.980	0.968	0.994
DT	0.981	0.992	0.997	0.993	0.982	1.000	0.992
Ensemble	*1.000*	1.000	1.000	1.000	1.000	1.000	1.000

Recall measures encompass the successful rate of non-depressive predictions and are almost equal to 1 in the experiment as displayed in Table 6, the ensemble classifier was successful in the prediction of non-depressive cases.

### Discussion

The ensemble classifier is superior to the baseline models in both F1 measure and Accuracy. It led the test results of both the overall dataset and all experiment results in sub-datasets as shown in Tables 4 and 5, respectively. It gathered different predictions from the baseline models and combined them into a better prediction. The ensemble proved more stable and robust than any involved baseline algorithm during the experiment. We utilised a random under-sampling technique with ensemble method to leverage the class imbalance problem where non-depression instances is about 10 times larger than depressed instances.

**Fig. 5:**
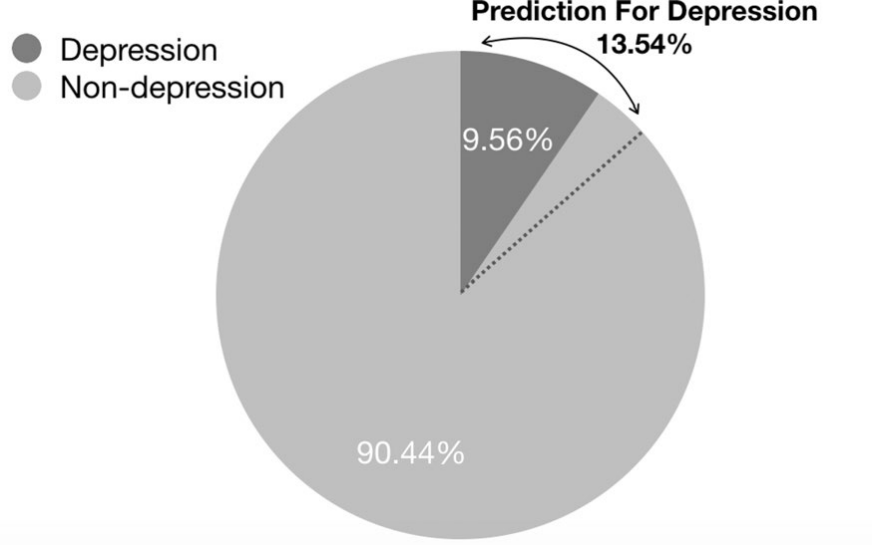
Coverage in correct classification of depression

The proposed ensemble method significantly improved predictive performance with class imbalance. By analysis of the performance in recall measure (see Table 6), the preferred ensemble method covers all depressed cases in PHQ-9 screening measurement where no depressed instance has been mistakenly labelled as non-depression. The recall performances of ensemble classifier is about 1 in the overall dataset and all sub-datasets. According to the definition of recall measure $Recall = \frac{TP}{TP + FN}$, it means that only when false negative measurement (FN) is 0, the recall measure is equal to 1. In our experiment, FN presents the number of depressed users who were incorrectly identified as non-depressed. As FN is zero, it indicates that no depressed instances in the experiment were mistakenly classified. The coverage in correct classification of depressed participants is perfect, only slightly larger than the results of the psychological screening (illustrated in Fig. 5). If we let the predicted precision be $P_{p}$ and percentage of non-depressed instances as $N_{1}$, the overall prediction $P_{o}$ of depressed instances is calculated as below:

5 $$\begin{aligned} P_{o} = 1 - (P_{p} \cdot N_{1})= 1 - 0.956 \cdot 90.44\% = 13.54\%. \end{aligned}$$

The coverage (see Fig. 5) of depression cases is slightly larger than the real situation of the MHI-5. However, it is acceptable for large sampling that there is no missing of any depression case and only about 4% of total cases have been incorrectly labelled as depressed in the prediction. The proposed ensemble method is perfect for preliminary screening of MDD cases for further clinical diagnosis without missing any potential depression case.

In comparison with the predictions in the different sub-datasets (see Table 3), the ensemble classifier performs the best in the overall dataset and has a similar accuracy in the mental sub-set. The importance of diagnostic criteria in mental symptoms demonstrates that mental criteria are the major features for identifying depression. Meanwhile, both accuracy and F1 measures for predicting depression in physical, social and role functionalities are equal to the predictive performance in general sub-set. The features in the mental sub-set have the most relevance to the conceptual framework of the classifier, which was expected given the domain knowledge used to build it. This demonstrated that even without clinical process and using the two-classification approach of depressive and non-depressive groups, the study still demonstrated that mental functionalities remain the most significant predictor for depression. This is most evident in the precision score, which was higher than the overall dataset precision shown in Table 3. However, while mental functionalities give reasonable accuracy and F1 score, adding other features from the QOLS increases the overall predictive performance of the model and shows that adding other life scales can assist in detecting depression. Mental features appear to be the most contributing factors which is consistent with psychological domain knowledge. In the overall dataset, further experiments might involve removing sub-sets from the overall dataset such as general features, which might impact the overall accuracy. Further study is needed on the features from the QOLS that have the strongest relationship with mental attributes.

Additional experiments adding other significant mental features to the sub-set and comparing it against the overall dataset might provide further insight into this correlation. The general sub-set (70 features) has more features than the physical (7 features), social (6 features) and role (9 features) sub-sets. This indicates that many features in the general sub-set occurred without enough specificity for classifying depressed and non-depressed labels. Partial general functional features hence are useless in the detection of depression. Correspondingly, features in physical, social and role functioning sub-sets are more correlational in the classification. Weak depression indicator is not only helpless in the classification, but also incline the overall predictive accuracy. Therefore, it is extremely critical for depression diagnostic approaches to select a limited number of suitable features to distinguish depressed cases from a wide range. From the result of this research, we suggest an algorithm for feature selection which first involves as more mental symptoms as possible according to depression diagnostic criteria and pluses no more than 50% features in health criteria in physical, role and social functionality. This algorithm ensures the majority of features consisted by mental diagnostic criteria and mixes partial health criteria to avoid the scenario that temporary mental status change occurs by sudden events like losing close relatives. It simulates the proceedings that psychologist did in the standard clinical interview.

## Conclusion and future work

This work presented a binary ensemble classifier which is able to distinguish depressive cases from non-depressive cases in a wide ranging health survey dataset. Importantly, the ensemble method does not miss any identification of potentially depressed case. In the experimental evaluation using the NHANES dataset, only 4% cases were mistakenly classified into depressed class and no depressed case were incorrectly detected. The ensemble classifier on the whole dataset has a high F1 measure of 0.976 compared to the PHQ-9 depression screen inventory, 95.4% and 95.6%, for Accuracy and Precision, respectively. It also demonstrated that the ensemble system is stable and robust for detecting depression on a partial dataset. The approach and the experiment also demonstrated that the combination of a classification technique with binary ground truth can provide stronger predictive performance compared to baseline standards. The ensemble method is very simple, close to the bagging and major voting ensemble methods. Other boost ensemble methods are also suggested to improve the prediction performance further, like the EUSBoost method [29].

Moving forward, this research presents a method that can assist in the preliminary screening of depressive cases in a large number of potential cases before formal clinical diagnosis. The significance is we demonstrate that an ensemble classifier outperforms baseline models in both distinguishing depressed and non-depressed cases, and predicting potential MDD diagnosis based on mental health severity scales in the PHQ-9 and health-related functionalities in the SF-20. With these two assessment instruments being widely used in healthcare, the system provides an efficient way to screen more people than traditional technologies and has a similar accuracy and coverage as the current PHQ-9. However, the reliability and sensitivity of this ensemble system need to be tested on additional datasets. In particular, adding additional features to the mental sub-set would provide further evidence about the importance of mental functionalities. Mental features appear to be the most significant contributing factors to predicting depression, however, further tests of these sub-sets in isolation and using relevant QOLS data will further improve the classification performance and understanding about the relationship between features and depression. Several possible future research directions for applying our ensemble method include using rich online social media sources to extract features for classification, as is the current trend in ML approaches [8]. Using this classification method on textual data will assist in improving the reliability and sensitivity of the ensemble system. Furthermore, deep learning techniques like DNN would increase the range of the ensemble classification. Hence, this will be our next work in improving this method.

## Data Availability

The authors would like to acknowledge the use of the National Health and Nutrition Examination Survey (NHANES) in the study and thank the Centers for Disease Control and Prevention of the Department of Health and Human Services, the United States for making the data set pubic for research purpose.
